# Literature Review and medication outcomes for CYP with dual diagnosis of ADHD and Autism in UK NHS CAMHS Service

**DOI:** 10.1192/j.eurpsy.2026.11145

**Published:** 2026-07-31

**Authors:** E. S. Lenku, S. Olety

**Affiliations:** 1 University of Manchester, manchester; 2 Tameside CAMHS, Stalybridge, United Kingdom

## Abstract

**Introduction:**

Attention Deficit Hyperactivity Disorder (ADHD) and Autism Spectrum Disorder (ASD) are common neurodevelopmental conditions that often co-occur, referred to as AuDHD. Their overlap presents distinct challenges in diagnosis, treatment, and management. Understanding this interplay is essential for effective intervention, particularly regarding pharmacological treatment within NHS services in the UK.

**Objectives:**

This study aimed to: (1) evaluate current treatment approaches for children and young people (CYP) with co-occurring ADHD and ASD, and (2) identify gaps in treatment protocols and medication outcomes within the NHS framework.

**Methods:**

A comprehensive literature review (2013-present) synthesised evidence on medication efficacy, side effects, and benefits for CYP with comorbid ADHD and ASD. A service evaluation was also conducted within Tameside and Glossop CAMHS, analysing records of CYP with ASD in the ADHD treatment pathway (March-August 2024). Data included medication types, dosage adjustments, side effects, and adherence.

**Results:**

Literature findings indicate that CYP with AuDHD often experience slower medication response and greater sensitivity to side effects compared with those with a single diagnosis. Emotional dysregulation is more pronounced, contributing to a wider range of adverse effects. Non-stimulant medications show promise in reducing cardiovascular side effects such as hypertension and tachycardia, while stimulant medications are generally more effective for ADHD alone. The service evaluation included 21 participants, 13 with dual diagnoses. Medication changes were mainly due to efficacy (93.7%), intolerance (21.5%), and non-adherence (6.5%), with emotional dysregulation and agitation commonly prompting adjustments.

**Conclusions:**

Results highlight the need for individualised treatment planning for CYP with dual diagnoses. Current NICE guidelines advise standard ADHD protocols to AuDHD; however, lower starting doses are recommended given increased medication sensitivity. Complementary non-pharmacological interventions, such as behavioural therapy and social skills training, are crucial to address complex emotional and behavioural needs.

Clinicians should adopt a holistic and cautious approach when treating CYP with AuDHD. Careful monitoring of irritability, agitation, aggression, and medical risks, such as seizures and tachycardia, is essential. Greater clinical awareness of emotional dysregulation and its impact on treatment outcomes is vital to ensuring safer and more effective care.

**Disclosure of Interest:**

None Declared

